# Regulation and Metabolic Significance of *De Novo* Lipogenesis in Adipose Tissues

**DOI:** 10.3390/nu10101383

**Published:** 2018-09-29

**Authors:** Ziyi Song, Alus M. Xiaoli, Fajun Yang

**Affiliations:** Departments of Medicine and Developmental and Molecular Biology, Albert Einstein College of Medicine, Bronx, NY 10461, USA; ziyi.song@einstein.yu.edu (Z.S.); alus.xiaoli@einstein.yu.edu (A.M.X.)

**Keywords:** adipocyte, *de novo* lipogenesis, transcription, post-translation, central regulation, ChREBP, SREBP, LXR, FASN, obesity, insulin resistance, thermogenesis

## Abstract

*De novo* lipogenesis (DNL) is a complex and highly regulated process in which carbohydrates from circulation are converted into fatty acids that are then used for synthesizing either triglycerides or other lipid molecules. Dysregulation of DNL contributes to human diseases such as obesity, type 2 diabetes, and cardiovascular diseases. Thus, the lipogenic pathway may provide a new therapeutic opportunity for combating various pathological conditions that are associated with dysregulated lipid metabolism. Hepatic DNL has been well documented, but lipogenesis in adipocytes and its contribution to energy homeostasis and insulin sensitivity are less studied. Recent reports have gained significant insights into the signaling pathways that regulate lipogenic transcription factors and the role of DNL in adipose tissues. In this review, we will update the current knowledge of DNL in white and brown adipose tissues with the focus on transcriptional, post-translational, and central regulation of DNL. We will also summarize the recent findings of adipocyte DNL as a source of some signaling molecules that critically regulate energy metabolism.

## 1. Introduction

Adipose tissues (AT), particularly white adipose tissues (WAT), are the major organ for energy storage [1]. WATs store extra energy from diets in the form of triglycerides (TG) or fat, which can be mobilized to meet energy demand in states of fasting or exercise. Meanwhile, ATs are also important endocrine organs. They secrete various adipokines such as leptin and adiponectin, and lipokines such as palmitoleate and fatty acid esters of hydroxyl fatty acids (FAHFA), to regulate systemic glucose and lipid metabolism [2,3,4,5]. Thus, AT dysfunction plays a pivotal role in the development of obesity and its associated diseases, including type 2 diabetes mellitus (T2DM), cardiovascular disease (CVD), non-alcoholic fatty liver disease (NAFLD), and several types of cancer [6,7,8,9]. Therefore, studies on ATs will provide opportunities to combat obesity-associated diseases [10,11].

Fat accumulation is determined by the balance between TG synthesis and breakdown. Upon feeding, fatty acids in ATs are from two distinct origins, that is, circulating TG and *de novo* lipogenesis (DNL) [12]. Circulating TGs are originally synthesized in the intestine or liver, and packaged into chylomicrons or very low density lipoproteins (VLDL), respectively. When those lipoproteins travel to ATs, TGs are hydrolyzed into non-esterified fatty acids (NEFA) by insulin-stimulated action of lipoprotein lipase (LPL) within vascular endothelium in ATs [13]. Released NEFAs enter adipocytes through fatty acid transporters such as CD36 and fatty acid transport protein-1 (FATP1) [14,15]. Meanwhile, insulin also stimulates adipocyte glucose uptake, which drives DNL in adipocytes. Fatty acids from these two sources are esterified using glycerol 3-phosphate derived from glucose as a backbone to form TG that is stored in lipid droplets.

During the periods of energy demand, that is, fasting or physical exercise, adipocytes mobilize stored fat to fulfill the energy need of other organs by lipolysis, in which each molecule of TG is broken down into three molecules of fatty acids and one molecule of glycerol. Three lipases act sequentially. First, adipose triglyceride lipase (ATGL) hydrolyzes TG into diacylglycerol (DAG) and the first molecule of fatty acid. Then, hormone-sensitive lipase (HSL) cleaves DAG into monoacylglycerol (MAG) and the second molecule of fatty acid. Ultimately, monoacylglycerol lipase (MGL) converts MAG into glycerol and the third molecule of fatty acid. These liberated fatty acids may be oxidized in muscle or brown adipose tissues (BAT), and glycerol may be used as a precursor for gluconeogenesis in the liver [16].

Under normal physiological conditions, lipogenesis and lipolysis are tightly and coordinately regulated by signals from peripheral tissues and the central nervous system, and both pathways are set into dynamic equilibrium to maintain fat content in ATs [17]. However, under pathological conditions, this equilibrium is disrupted. Consequently, unrestrained WAT lipolysis results in increased fatty acid release, leading to lipotoxicity and insulin resistance [18], while impaired lipogenesis in WAT decreases the synthesis of insulin-sensitizing fatty acid species, which also leads to insulin resistance [19]. As adipocyte lipolysis has been recently reviewed [16,20,21,22], here we focus on adipocyte lipogenesis and emphasize the recent progress in this field.

## 2. *De Novo* Lipogenesis (DNL)

**Figure 1:**
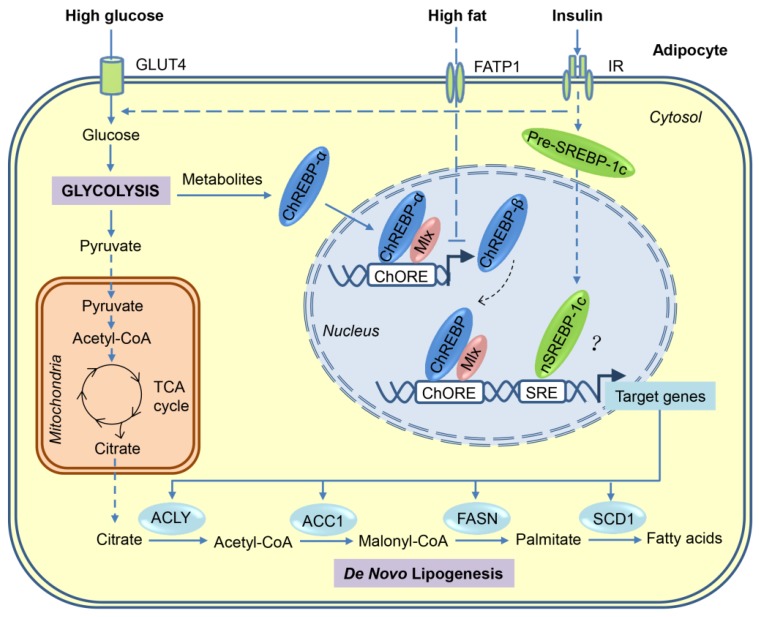
Transcriptional activation of *de novo* lipogenesis in adipocytes in response to high-sugar or high-fat diets. After the consumption of carbohydrates, a portion of the circulating glucose is taken by adipocytes through insulin-stimulated GLUT4, and then through glycolysis in the cytosol, glucose is converted to pyruvate, which is transported into mitochondria for further oxidation in the tricarboxylic acid (TCA) cycle. Citrate, an intermediate of the TCA cycle, is exported into cytosol and used as a substrate for *de novo* lipogenesis. Regulation of lipogenesis is mainly at the transcriptional level and carbohydrate response element-binding protein (ChREBP) plays a major role in adipocyte lipogenesis. Glucose metabolites generated during glycolysis activate ChREBP-α, which, together with Max-like protein X (MLX), binds to the carbohydrate response elements (ChoRE) in the promoters of target genes, including those encoding ATP-citrate lyase (ACLY), acetyl-CoA carboxylases 1 (ACC1), fatty acid synthase (FASN), stearoyl-CoA desaturase-1 (SCD1), and ChREBP-β. The induced ChREBP-β in turn further activates its target gene expression, which ultimately promotes the synthesis of fatty acids. Another lipogenic transcription factor sterol regulatory element-binding protein-1 (SREBP-1), induced by insulin at multiple levels, may play a minor role in adipocyte lipogenesis. Compared with carbohydrates, fat consumption inhibits *de novo* lipogenesis in adipocyte mainly through blocking the activation of ChREBP-β. FATP—fatty acid transport protein-1; IR—insulin receptor.

Carbohydrates can be converted to fatty acids through the process of DNL. When energy is excessive in the body, most of the newly synthesized fatty acids are esterified to become TGs for storage. As shown in Figure 1, a series of coordinated enzymatic reactions are involved in the flow of carbons from glucose to fatty acids [23,24]. First, glucose derived from dietary carbohydrates undergoes glycolysis and tricarboxylic acid (TCA) cycle to produce citrate in the mitochondria, which is transported to cytosol and then releases acetyl-CoA by ATP-citrate lyase (ACLY). Second, the resulting acetyl-CoA is converted to malonyl-CoA by acetyl-CoA carboxylases 1 (ACC1). Third, fatty acid synthase (FASN), the key rate-limiting enzyme in DNL, converts malonyl-CoA into palmitate, which is the first fatty acid product in DNL. Finally, palmitate undergoes the elongation and desaturation reactions to generate the complex fatty acids, including stearic acid, palmitoleic acid, and oleic acid.

In principle, DNL takes place in all cells given the fact that fatty acids are the structural elements of cell membranes, but it is more active in metabolic tissues, such as liver, ATs, and skeletal muscle [25]. In rodents, liver is the major contributor to the whole-body lipogenesis, and ATs contribute much less than that of the liver. However, studies in humans fed with a carbohydrate-rich diet revealed that total fat synthesis in ATs significantly exceeded hepatic DNL [26], suggesting that ATs may be the second major site for fat synthesis. In particular, recent studies show that adipocytes generate adipocyte-specific fatty acids that act to improve systemic insulin sensitivity and decrease inflammation [27,28]. Therefore, adipocyte DNL is an important source of endogenous fatty acids and plays key roles in maintaining systemically metabolic homeostasis. Of note, although normally DNL in skeletal muscle is not a major contributor of total fatty acid flux in this tissue, it is induced under high-fat diet conditions [29]. Inhibition of DNL by skeletal muscle-specific FASN deletion improves systemic insulin sensitivity without altering adiposity, but decreases muscle strength [29], suggesting that DNL also plays important roles in skeletal muscle, especially in states of insulin resistance.

In addition to measuring lipogenic gene expression, several approaches have been used to trace fatty acid synthesis. One of the earliest approaches involved the use of radiolabeled substrates (e.g., ^14^C-acetate) to measure isotopic enrichment in the lipid fraction. The advantage of this approach is that it requires minimal sample preparation and no mass spectrometry. The downside, however, is that it is not specific to particular fatty acids, and thus does not provide information on isotope enrichment per molecule [30]. To overcome this shortage, approaches with increased specificity have been developed by combining stable isotope tracers with mass spectrometry analysis [31]. In this updated approach, deuterated water (D_2_O) is often used in cultured cells or animals. The principle of this approach is that the ^2^H in D_2_O is incorporated into fatty acids during DNL and the degree of incorporation is directly proportional to the rate of biosynthesis. Using these approaches, DNL is directly studied.

DNL is highly controlled by hormones and nutritional status. During fasting, DNL is low, owing to increased blood glucagon and cellular cAMP levels, which inhibit DNL through activating AMP-activated protein kinase (AMPK) [32,33] and cAMP-dependent protein kinase (PKA) [34,35]. By contrast, after a carbohydrate-rich meal, blood glucose and insulin levels rise, which stimulate DNL through increasing the substrate availability, lipogenic enzymes activity, and lipogenic genes expression. It is noteworthy that food composition also has dramatic effects on DNL in ATs. For instance, fructose- or sucrose-rich diets strongly induce DNL in both liver and ATs. In contrast, high-fat diets significantly inhibit DNL [36,37].

Importantly, mammals have a limited capacity to store energy in the forms of carbohydrates, but are able to store seemingly unlimited amounts of TGs. Therefore, DNL plays a key role in the integration of glucose and lipid homeostasis. Dysregulation of DNL contributes to many metabolic problems, including hyperglycemia, hyperlipidemia, insulin resistance, T2DM, NAFLD, and CVD [23,38,39].

## 3. Transcriptional Regulation of DNL in Adipocytes

Many of the enzymes involved in DNL are regulated primarily at the transcriptional level in a coordinated manner. Transcriptional activation of these lipogenic genes after a carbohydrate-rich meal can be achieved through the complex mechanisms involving multiple transcription factors. Using loss/gain-of-function approaches, sterol regulatory element-binding protein (SREBP)-1, carbohydrate response element-binding protein (ChREBP), and liver X receptors (LXRs) are identified as major lipogenic transcription factors in liver in response to insulin, glucose, and polyunsaturated fatty acids, respectively. However, only ChREBP seems to be the major driver for adipocyte DNL (Figure 1). The relevant data are also summarised in Table 1 and Table 2.

### 3.1. SREBP-1

The SREBPs are a family of membrane-bound transcription factors that were identified as important regulators of cholesterol and fatty acid homeostasis [40]. In mammals, there are three SREBP isoforms (i.e., SREBP-1a, SREBP-1c, and SREBP-2) encoded by two different genes, named *Srebf*1 and *Srebf*2. SREBP-1a and SREBP-1c are derived from *Srebf*1 by alternative splicing of the first exon. With a longer transactivation domain, SREBP-1a is transcriptionally more potent than SREBP-1c [41]. The third SREBP isoform, SREBP-2, is transcribed from a different gene *Srebf*2, but has a low homology with SREBP-1a/c [42]. Functionally, SREBPs activate distinct but overlapping programs in lipid metabolism. SREBP-1a activates both fatty acid and cholesterol synthesis, and SREBP-1c only induces fatty acid synthesis, whereas SREBP-2 is primarily responsible for cholesterol synthesis and uptake. Of the three SREBPs, SREBP-1c is more abundant in tissues with active DNL such as liver and ATs, and its function is predominantly regulated by insulin at multiple regulatory steps, including transcription, post-translation, and protein stability [43,44,45].

Transcriptionally, the *Srebf1c* mRNA is strongly induced by insulin via a mechanism involving the LXR transcription factors, as well as SREBP-1c feed-forward activation [46,47]. However, like SREBP-2, SREBP-1c is synthesized as an inactive membrane-bound precursor in the endoplasmic reticulum (ER). Maturation of SREBP-1c involves transportation of the precursor by the SREBP cleavage-activating protein (SCAP) from ER to Golgi, where the inactive precursor is cleaved sequentially by two proteases S1P and S2P [48]. The N-terminal fragment of SREBP-1c is then translocated into the nucleus and activates lipogenic gene expression. This is a highly regulated process, in which insulin plays a key role primarily through the activation of canonical PI3K/AKT pathway and the mammalian target of rapamycin complex 1 (mTORC1) [49]. A recent study shows that Per-Arnt-Sim (PAS) kinase is also required for SREBP-1c maturation and activation [50]. The mature/nuclear/active form of SREBP-1c is very unstable, and several factors are involved in the regulation of its protein stability. Insulin stabilizes the mature SREBP-1c through glycogen synthase kinase-3β (GSK-3β) inhibition [51] and Lipin1 phosphorylation [52]. Moreover, our group found that CDK8, a subunit of the conserved Mediator complex that is down-regulated by insulin in the liver, negatively regulates the mature SREBP-1c protein stability by enhancing SREBP-1c phosphorylation and thus protein degradation [53].

Accumulating evidence suggests that SREBP-1c is a major regulator of hepatic DNL as it is both sufficient and necessary for DNL in the liver [54]. For example, pathological increase of SREBP-1c or genetic overexpression of SREBP-1c in the liver causes fatty liver [55,56]. Conversely, pharmacological inhibition or genetic inactivation of SREBP-1c protects from the development of fatty liver [57,58]. However, SREBP-1c seems to be a minor player for DNL in ATs. Although SREBP-1c is also both sufficient and necessary to promote lipogenic enzyme expression in adipocytes in vitro [59], global SREBP-1 knockout mice displayed normal mRNA levels of lipogenic enzymes in WATs when fed with the normal chow [60]. Moreover, despite the amelioration of fatty liver, loss of SREBP-1 in genetically obese *ob*/*ob* mice also did not affect the lipogenic gene expression in WATs [57]. Interestingly, under the caloric restriction condition, SREBP-1c deficiency seemingly inhibits caloric restriction -induced upregulation of lipogenic genes in WATs, but not in the liver [61]. These loss-of-function studies suggest that SREBP-1c in adipocytes may not be important for DNL in vivo. One explanation is that the effect of SREBP-1 deficiency on DNL is compensated by other factors, particularly SREBP-2 [60]. However, a key caveat of those studies is that the animal models were global SREBP-1-deficient. To precisely define the importance of SREBP-1c in adipose tissues, adipocyte-specific SREBP-1c-deficient mouse models, that is, SREBP-1c knockout in Adiponectin (*Adipq*)-positive cells, are required in the future.

In addition, it has been reported that an increase of SREBP-1c expression in WATs by the treatment of LXR agonist T0901317 is not accompanied by the up-regulation of lipogenic genes such as FASN, ACC1, and stearoyl-CoA desaturase-1 (SCD1) [62]. Consistent with the gene expression, SREBP-1c was neither recruited to the *Fasn* promoter nor did it induce the activity of a *Fasn* promoter-driven reporter gene in adipocytes [62]. Moreover, SREBP-1c transgenic mice driven by the aP2 (*Fabp*4) promoter also did not display an increase of lipogenic gene expression in WATs, although some lipogenic genes such as *Fasn*, *Acc*1, and *Scd*1 were up-regulated in BAT [63]. However, it is unclear whether these effects are direct or indirect because the transgenic mice also exhibit impaired adipocyte differentiation, severe AT lipodystrophy, insulin resistance, and fatty liver [64]. Together, these gain-of-function studies also argue against SREBP-1c as a major driver for adipocyte DNL. By contrast, fat-specific overexpression of SREBP-1a, which is much lower in abundance than SREBP-1c in ATs, significantly increased lipogenic genes expression and fatty acid synthesis in both WAT and BAT, leading to ATs hypertrophy [63]. This result suggests that SREBP-1a and SREBP-1c have distinct roles in adipocyte fat metabolism in vivo. Nonetheless, a caveat of those studies is that the aP2 promoter is active not only in adipocytes, but also in macrophages [65]. As macrophages in ATs are known to play a regulatory role in metabolism [66,67], the aP2 promoter used in these studies may have complicated the results. Therefore, future studies using adipocyte-specific promoters to overexpress SREBP-1c may be necessary.

### 3.2. ChREBP

ChREBP, also known as MLXIPL or MONDOB, is a member of basic helix–loop–helix/leucine zipper transcription factor family that is responsible for carbohydrate-induced transcription of glycolytic and lipogenic enzymes [68,69]. It has two isoforms, ChREBP-α and the recently identified ChREBP-β, which is encoded by the same *Mlxipl* gene, but through the use of alternative promoters [70].

ChREBP-α is constitutively expressed in metabolically-active tissues, such as liver, adipose tissues, skeletal muscle, intestine, kidney, and pancreas [68], but during fasting or under low glucose conditions, ChREBP-α was phosphorylated by PKA [35] and AMPK [33] at multiple sites to retain it in the cytosol. During feeding or high glucose conditions, the intermediates of glucose metabolism, such as xylulose 5-phosphate or glucose 6-phosphate, activate ChREBP-α through multiple insulin-independent mechanisms, including dephosphorylation, nuclear translocation, protein–protein interactions, and release of the transactivation domain inhibition [71,72,73]. Upon activation, ChREBP-α forms a heterodimer with MLX and induces the expression of genes primarily involved in glycolysis, DNL, and fatty acid desaturation [68,74]. In contrast, ChREBP-β isoform, which lacks most of the N-terminal low glucose-inhibitory domain, is constitutively active in stimulating target gene expression, and its transcription is induced in a feed-forward manner by ChREBP-α and itself (Figure 1) [70]. To date, other regulators that control the expression of ChREBP-β remain unknown.

In agreement with its function in the liver (reviewed by authors of [75]), ChREBP is also a major determinant of fatty acid synthesis in ATs. ChREBP-α is highly expressed in both WAT and BAT in human, mouse, and rat. It is also expressed in preadipocytes, and its level increases dramatically during differentiation of human and mouse preadipocytes [76,77]. Overexpression of a constitutively active ChREBP in mouse 3T3-L1 white preadipocytes increases lipogenic gene expression and promotes adipocyte differentiation [77]. Conversely, reducing endogenous ChREBP activity impairs adipocytes differentiation [77]. Mechanistically, it is suggested that some unknown fatty acid derivatives from ChREBP-mediated DNL are required for the activation of nuclear receptor peroxisome proliferator-activated receptor (PPARγ), the master transcription factor in adipogenesis [77]. In support of this model, supplement of the PPARγ ligand, rosiglitazone, can completely rescue the differentiation defect caused by ChREBP deficiency, including adipogenic and lipogenic marker genes expression and lipid accumulation [19].

Consistent with the in vitro results, overexpression of constitutively active ChREBP isoform in adipose tissues increased the expression of genes involved both in DNL such as *Fasn*, *Acly*, *Acc*1, *Scd*1, and *Elovl*6, and adipocyte differentiation such as *Pparg*2, *Cebpa*, and *Fabp*4 [78]. Conversely, mice with global ChREBP deficiency displayed significant impairment of lipogenic gene expression and hepatic DNL; these mice are intolerant to simple carbohydrates and develop insulin resistance [68]. Although the authors did not examine DNL in fat, the weights of both WAT and BAT from ChREBP-deficient mice were dramatically reduced [68], suggesting that adipocyte DNL is probably also impaired. This speculation was confirmed by a recent study, in which adipocyte-specific ChREBP-mutant mice were studied, and loss of ChREBP in fat dramatically impaired sucrose-induced lipogenic gene expression and DNL in both WAT and BAT, but not in the liver [19]. However, compared with global ChREBP-deficiency, adipocyte-specific ChREBP-deficiency had little effect on the weights and sizes of adipose tissues, regardless of whether the mice were fed with the normal chow diet or high-fat diet [19,68]. This is probably because of the compensatory effects of increased fatty acids uptake from the circulation. Although further studies are needed, it is clear that unlike SREBP-1c, ChREBPs are both necessary and sufficient to drive DNL in adipocytes.

Consistent with the conclusion that ChREBPs are the major lipogenic transcription factors in ATs, the upstream lipogenic signals or other lipogenic factors in adipocyte regulate DNL directly or indirectly through the regulation of ChREBP expression or transcriptional activity. GLUT4 (also known as SLC2A4), the major glucose transporter in adipocytes, determines the activity and expression of ChREBPs through the regulation of glucose uptake. Adipocyte-specific overexpression of GLUT4 increased lipogenic enzyme expression, fatty acid synthesis, lipid accumulation, and adiposity through ChREBP-α-mediated induction of ChREBP-β expression, but not through SREBP-1c [70]. Conversely, Adipocyte-specific knockout of GLUT4 resulted in opposite effects [70]. mTORC2 is a master regulator of metabolism and controls DNL in both WAT [37] and BAT [79]. Conditional deletion of the essential mTORC2 subunit RICTOR in mature adipocytes reduced ChREBP-β expression and DNL in WAT partially through the downregulation of GLUT4-mediated glucose uptake [37]. Intriguingly, inhibiting adipocyte lipid uptake by conditional deletion of LPL resulted in a compensatory increase of DNL in both WAT and BAT owing to the up-regulation of the GLUT4/ChREBP-β pathway [80]. More recently, the serine/threonine-protein kinase AKT2, an effector molecule in the insulin signaling pathway, has been identified as a cold-induced kinase in BAT that is required for adipocyte DNL by stimulating the ChREBP-β transcriptional activity [36]. Collectively, these studies strongly suggest that the ChREBP transcription factors are the major activators of DNL in adipocytes.

### 3.3. LXRs

The oxysterol-activated nuclear receptor LXRs, that is, LXRα and LXRβ, were initially characterized as key regulators of hepatic cholesterol and lipid metabolism [81]. LXRα and LXRβ are encoded by two different genes, but share a considerable sequence homology [82]. LXRα is expressed primarily in liver, ATs, intestine, and macrophages [83], whereas LXRβ is ubiquitously expressed [84]. Upon binding to ligands, LXRs undergo a conformational change that promotes interaction with coactivators to facilitate transcription of target genes. Like other nuclear receptor family members, LXRs are modulated by a wide range of post-translational modifications, including SUMOylation, phosphorylation, acetylation, ubiquitination, and O-GlcNAcylation [85]. For example, under fasting condition, hepatic LXRα is directly phosphorylated by PKA, which impairs LXRα DNA binding activity and inhibits the expression of its target genes such as SREBP-1c [86].

The functions of LXRs have been well studied in the liver. They stimulate hepatic DNL by direct activation of the promoters of lipogenic genes such as *Srebf1c* and *Fasn* [47]. Activation of LXRs in mice by oral administration of a synthetic LXR agonist T0901317 led to a marked increase in SREBP-1 and lipogenic enzyme expression, as well as TG content in the liver [46]. In agreement with this study, another study reported that mice developed fatty liver in three weeks after the treatment with T0901317 by intraperitoneal injection under high-fat diet conditions [87]. Furthermore, global loss of LXRs in *ob*/*ob* mice impairs hepatic lipogenesis and reduces hepatic steatosis compared with control because of the decreased expression of SREBP-1 and lipogenic enzymes [88].

However, the published data suggest that LXRs may target different pathways in adipocytes. In contrast to those results from the liver, global loss of LXRs in *ob*/*ob* mice significantly enhanced lipogenesis and adipogenesis in ATs, leading to enlarged fat tissues and improved insulin sensitivity [88]. This is probably owing to the up-regulation of the PPARγ and GLUT4/ChREBP-β pathways [88]. In agreement with this report, adipose tissue-specific LXRα knockout mice also had severe adiposity with a concomitant increase in fat mass and adipocyte size when fed with a high-fat diet [90]. The underlying mechanism is probably that LXRα deficiency in fat impairs adipocyte lipolysis and fatty acid availability and oxidation [90]. Conversely, administration of LXRα agonist T0901317 in mice fed with a high-fat diet reduced fat mass, which was accompanied with increased adipocyte lipolysis and apoptosis, and decreased PPARγ transcriptional activity [87]. Although different mouse models and diets may contribute to the different effects of LXRs in ATs, these studies suggest that LXRs play a different role in adipocytes and hepatocytes. Nonetheless, it is less likely that LXRs are major regulators of DNL in adipocytes.

## 4. Post-Translational Regulation of DNL in Adipocytes

As discussed above, most enzymes involved in DNL are primarily regulated at the transcriptional level, however, the activities or protein stability of these enzymes are also regulated at the post-translational level. During fasting, the lipogenic enzymes are restrained at low activities. In response to feeding, their enzymatic activities are acutely increased by post-translational modifications, including phosphorylation and O-GlcNAcylation.

Phosphorylation is a common post-translational modification of proteins. Recently, it has been reported that phosphorylation of ACLY, the first enzyme in DNL, is dynamically regulated by the hepatic branched chain alpha-keto acid dehydrogenase kinase (BDK) and protein phosphatase Mg^2+^/Mn^2+^ -dependent 1K (PPM1K) [93], which are previously known to control branched-chain α-ketoacid dehydrogenase (BCKDH) activity and branched-chain amino acids (BCAA) levels. Phosphorylation of ACLY on Ser454 by BDK increases the ACLY activity for the generation of acetyl-CoA and subsequent malonyl-CoA, eventually promoting hepatic DNL. Importantly, this modification is physiologically regulated during the fasting-feeding cycle by ChREBP-β, which up-regulates BDK expression and concomitantly inhibits PPM1K expression during feeding [93]. Thus, after a carbohydrate-rich meal, ChREBP not only directly stimulates the *Acly* gene transcription, but also enhances the activity of ACLY through modulating the ratio of BDK to PPM1K. However, so far it is still unclear whether ACLY in adipocytes is also regulated by BDK and PPM1K. In contrast, phosphorylation inhibits the activities of ACC1 and FASN. The serine/threonine kinase AMP-activated protein kinase (AMPK) acts as a major energy sensor and regulator in ATs [94]. When activated by intracellular energy depletion, nutrient deprivation, or hypoxia, AMPK phosphorylates ACC1 at Ser79 and FASN at unknown site(s) in adipocytes, leading to direct inhibition of the production of malonyl-CoA and palmitate, respectively. Consequently, DNL is inhibited by the activation of AMPK [95,96,97]. O-GlcNAcylation is a highly dynamic post-translational modification, which is controlled by two antagonistic enzymes: O-Linked N-Acetylglucosamine (O-GlcNAc) transferase (OGT), which transfers the GlcNAc group onto serine or threonine residues of protein substrates [98]; and O-GlcNAcase (OGA), which removes the sugar moiety from substrates [99]. A recent study reported that FASN is directly modified by O-GlcNAcylation in the liver [100]. Elevating O-GlcNAcylation of FASN by glucose-induced activation of OGT or drug-targeted inhibition of OGA increases the interaction between FASN and ubiquitin-specific protease-2a (USP2A), which acts to remove ubiquitination and is known to stabilize FASN [101]. As a result, O-GlcNAcylation of FASN leads to accumulation of this enzyme and DNL in the liver [100]. Although O-GlcNAcylation of FASN in adipocytes has not been reported to date, given the fact that O-GlcNAc modification is involved in development of insulin resistance and glucose-toxicity in adipocytes [102,103], it is possible that adipocyte FASN is also regulated by O-GlcNAcylation. However, whether other lipogenic enzymes are regulated by O-GlcNAcylation or other modifications such as acetylation is currently unclear and deserves to be investigated in the future.

## 5. Central Regulation of DNL in Adipocytes

A growing number of studies have demonstrated that adipocyte DNL is not only regulated by the peripheral signals, but also by the central nervous system [104,105]. To date, insulin and leptin are two reported signals in medial basal hypothalamus (MBH) that play a key role in maintaining DNL in WATs [106].

Insulin has a direct effect on adipocyte DNL through the insulin receptor, which leads to the activation of PI3K-AKT signaling pathway and inhibition of PKA [43]. However, studies also point to an indirect role of insulin on DNL through MBH [107]. When the insulin level is acutely raised in the brain by infusing insulin directly into MBH of rats, the expression of lipogenic enzymes including *Fasn* and DNL in WATs were increased, while the activity of HSL and adipocyte lipolysis were suppressed [107]. Conversely, mice lacking the neuronal insulin receptor exhibit decreased DNL and unrestrained lipolysis in WATs [107]. Mechanistically, MBH insulin signals likely inhibit sympathetic outflow to WATs, as surgical denervation or pharmacological sympathectomy abolishes the effects of brain insulin on WAT lipogenesis and lipolysis [107]. In contrast, leptin, an adipokine that plays a key role in the control of body weight through regulating food intake and fuel partitioning, was found to exert an effect opposite to insulin in MBH [108]. Acute infusion of leptin into MBH of rats inhibits lipogenic enzyme expression and DNL, but activates lipolysis in WATs [108]. This is probably because the MBH leptin signaling is able to reduce the endocannabinoid anandamide level in WATs via sympathetic innervation [108]. It has been shown that anandamides can stimulate lipogenesis by activating the cannabinoid receptors [109]. Supporting this mechanism, when the rats were treated with the cannabinoid receptor agonist Win 55,212-2, MBH leptin failed to suppress DNL in WATs [108]. Furthermore, surgical denervation also blocks the inhibitory effect of MBH leptin on WAT lipogenesis [108]. Taken together, MBH insulin stimulates DNL and suppresses lipolysis in WATs, whereas MBH leptin acts oppositely, and these effects are mediated through either stimulation or inhibition of the sympathetic outflow to WATs [106,107,108].

Apart from insulin and leptin, a newly-identified neuropeptide, neurosecretory protein GL (NPGL), is reported to be involved in adipocyte DNL [110]. NPGL infusion or overexpression in MBH induced DNL specifically in WATs, but not in liver, and also increased the size of white adipocytes and adiposity [110]. Conversely, administration of neutralizing antibody against NPGL decreased white adipocyte size [110]. However, unexpectedly, NPGL mRNA expression is induced by fasting and inhibited by insulin [110]. As both insulin and NPGL promote adipocyte DNL, insulin inhibition of NPGL expression may constitute a negative feedback mechanism in MBH to curb adipocyte DNL in order to avoid adipocyte hypertrophy. To date, it remains unclear whether there are other signals in the central nervous system that can regulate adipocyte DNL.

## 6. Role of Adipocyte DNL in Insulin Resistance

A number of studies indicate that DNL in ATs has both direct and indirect beneficial effects to the body [111]. An increase in DNL promotes the conversion of excessive carbohydrates into fatty acids for energy storage. This pathway is of physiological importance especially when the diet is rich in carbohydrates, because hyperglycemia causes cellular damages and organ dysfunctions due to glucotoxicity [112,113]. Therefore, DNL helps to maintain glucose homeostasis. Moreover, adipocyte DNL is involved in the regulation of systemic glucose and lipid metabolism through the generation of lipokines [5,27].

### 6.1. DNL in White Adipocytes

**Figure 2:**
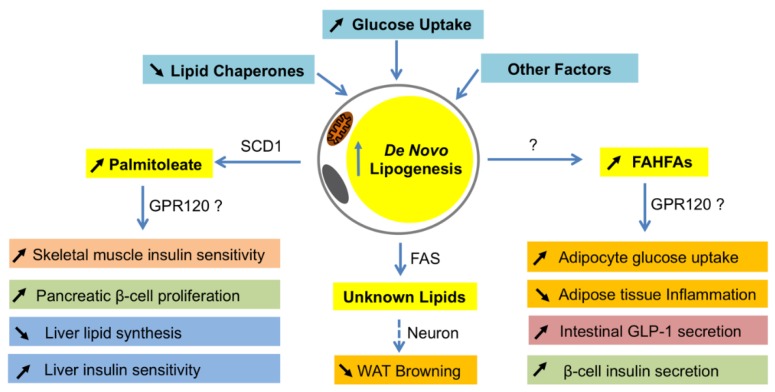
Systemic effects of lipokines produced by *de novo* lipogenesis in adipocytes. When *de novo* lipogenesis is increased in adipocytes by means of increasing glucose uptake, decreasing lipid chaperones, or others, some bioactive fatty acids such as palmitoleate and fatty acid ester of hydroxyl fatty acids (FAHFAs) are produced. As a product of SCD1 in adipocytes, palmitoleate functions to improve insulin sensitivity in skeletal muscle and liver, promote pancreatic β-cell proliferation, and inhibit lipid synthesis in the liver. Although it is unclear how FAHFAs are synthesized in adipocytes, these lipids have a function to stimulate adipocyte glucose uptake, intestinal glucagon-like peptide-1 (GLP-1) secretion and β-cell insulin secretion, and reduce inflammation in adipose tissues. The metabolically beneficial effects of palmitoleate and FAHFAs are probably through G protein-coupled receptor 120 (GPR120). In addition, FASN may produce some unknown lipid products in white adipocytes that function to inhibit white fat browning through neuronal circuit regulation. WAT—white adipose tissues.

Different from the changes in the liver, DNL is usually decreased in fat when animals or humans are obese or insulin resistance [114,115,116,117]. Based on the available data, such reduction seems to be a causative factor for the development of insulin resistance. For example, a recent study shows that adipocyte-specific reduction of DNL by ChREBP knockout causes insulin resistance and inflammation [19]. Therefore, adipocyte DNL is important for maintaining systemic insulin sensitivity. Interestingly, these beneficial effects of DNL in fat are found to be exerted by producing insulin-sensitizing fatty acids, such as palmitoleate and FAHFAs (Figure 2) [118,119].

Palmitoleate, a 16-carbon monounsaturated fatty acid, is produced mainly in adipocytes by FASN-mediated synthesis of palmitate, followed by SCD1-catalyzed desaturation [120]. Palmitoleate was first described as an insulin-sensitizing lipokine when the researchers examined the mice with a combined loss of fatty acid-binding proteins FABP4/aP2 and FABP5 [118]. Those mice were protected from high fat diet-induced obesity and fatty liver, and displayed profound insulin sensitivity and altered lipid profiles [118]. To determine if a specific lipid was correlated with the insulin sensitivity in those mice, an unbiased lipidomics approach was used to examine the lipid profiles of WAT and identified palmitoleate (C16: 1*n*7), which was the most significantly elevated lipid in adipose tissues, as well as serum. When wildtype mice were treated with palmitoleate, their muscle insulin sensitivity was increased while hepatic DNL was suppressed, suggesting that palmitoleate functions as a circulating lipokine and has insulin sensitizing effects [118]. Supporting the metabolic role of palmitoleate, G protein-coupled receptor 120 (GPR120) knockout mice had a reduced SCD1 expression in adipose tissues and lower levels of palmitoleate in plasma and adipose tissues, and those animals displayed lower insulin sensitivity with reduced phosphorylation of the insulin receptor, insulin receptor substrates, and protein kinase B in muscle, liver, and fat [74]. Subsequent studies in rodents further uncovered more fascinating roles of palmitoleate in metabolism (reviewed in the works of [121,122]).

However, the data on palmitoleate in humans are mixed. One study reported that circulating palmitoleate levels strongly and independently predict the insulin sensitivity of individuals at the high risk for T2DM [123]. In addition, human primary adipocytes extracted from the gluteofemoral depot showed higher production and release of palmitoleate compared with those from the abdominal depot, which was correlated with better insulin sensitivities and higher *Scd*1 expression [75]. Whereas, another study found that decreased palmitoleate in plasma and in VLDL is not associated with insulin resistance in skeletal muscle, liver, or adipose tissues in obese people [124]. Moreover, it is reported that palmitoleate is not related to insulin sensitivity in type 1 diabetes [125]. These discrepancies are probably the result of the analyses of different lipid fractions from different tissues or diseases. Nevertheless, palmitoleate represents an important link between adipocyte DNL and systemic insulin resistance.

Fatty acid esters of hydroxy–fatty acids or FAHFAs are another molecular link between adipocyte DNL and insulin resistance. Studies of adipocyte-specific GLUT4 transgenic (AG4OX) mice revealed that despite being obese and having elevated circulating fatty acids, those mice were more glucose tolerant and DNL in fat was elevated [119]. Lipidomic analysis of WATs from AG4OX mice led to the discovery of a new class of lipids, called FAHFAs, which were elevated in WATs and serum of AG4OX mice, and have beneficial effects in metabolism [119] (reviewed by authors of [126]). The most abundant forms of FAHFA in serum and fat of AG4OX mice are palmitic acid ester of hydroxyl stearic acids (PAHSA), and of which 9-PAHSA is the most abundant and biologically active isomer in adipose tissues [119]. Treatment of obese mice with 9-PAHSA lowers blood glucose and improves glucose tolerance, while stimulating insulin secretion in pancreas and glucagon-like peptide 1 secretion in intestinal cells, as well as insulin-stimulated glucose transport in adipocytes [19]. Inflammatory cytokine production from immune cells and ameliorate adipose inflammation in obesity is also reduced by 9-FAHFA [119]. The levels of FAHFAs are dynamically regulated by physiological and pathophysiological conditions. Fasting and high-fat diet feeding in mice alters the levels of FAHFAs in fat and serum in a tissue-specific and/or isomer-specific manner [119]. Interestingly, serum FAHFA levels are highly correlated with insulin sensitivity in humans [119]. Although the functions of FAHFAs in humans are still unclear, they represent another important link between adipocyte DNL and systemic insulin sensitivity.

The synthesis of FAHFAs in adipocytes is induced by the GLUT4/ChREBP-mediated DNL. First, the expression of GLUT4 and ChREBP-β in ATs are also reduced in obese mice and humans, and highly correlated with insulin sensitivity in humans [70,111]. Second, the detrimental effects of adipocyte-specific ChREBP knockout, in which PAHSAs were reduced, on insulin sensitivity and inflammatory responses can be completely abolished by 9-PAHSA supplementation [19]. Third, adipocyte-specific overexpression of ChREBP-β improved insulin sensitivity and reduced inflammatory genes expression in response to a Western diet [78]. Of note, the production of PAHSAs and palmitoleate may be regulated in different manners, as in RICTOR-knockout mice, the GLUT4/ChREBP-mediated DNL was inhibited, but somehow palmitoleate was elevated in WATs [37]. This is probably a compensatory response to the reduction of PAHSAs. Although the molecular mechanisms underlying the insulin-sensitizing effects of PAHSAs remain unclear, GPR120 seems to be involved [119].

It is unclear whether there are other lipokines that also exert an insulin-sensitizing effect, and how these lipokines may act and interplay in states of insulin resistance. Future studies are necessary to address these important questions. Interestingly, the metabolic benefits of elevated DNL in adipocytes may depend on how DNL is induced. For example, specific inhibition of fatty acids uptake in ATs by genetic ablation of LPL, the master regulator of fatty acid uptake from triglyceride-rich lipoproteins, caused a profound increase of DNL in both WAT and BAT, a reduction of adiposity, and an improved profile of blood insulin and adipokines [80]. However, neither glucose tolerance nor inflammatory markers were ameliorated in those mice [80]. One possible explanation is that loss of LPL potentially eliminates certain essential fatty acids from diets [80]. This may counteract the beneficial effects of increased DNL in adipocytes.

### 6.2. DNL in Brown Adipocytes

Although BAT has a higher rate of DNL than WAT [19], and FAHFAs are also synthesized in BAT [118], the contribution of DNL in BAT to whole body insulin sensitivity seems to be limited. A recent study has shown that inhibition of ChREBP-mediated DNL in BAT by AKT2 knockout remodeled the global lipid landscapes and reduced lipid content in BAT, but glucose and insulin tolerance as well as body mass were not affected [36]. Consistent with this study, BAT-specific knockout of FASN also did not affect glucose tolerance under both normal chow and high-fat diet conditions [114]. One possibility is that the BAT mass is quantitatively much less than that of WATs so that BAT contributes only a small portion of total beneficial lipokines in the body. Another possibility is that lipokines produced by BAT may be different from those by WATs. Recent studies identified a BAT-specific lipokine, 2,13-diHOME, which promotes fatty acid uptake by BAT and skeletal muscle in response to cold and exercise, respectively [127,128]. Notably, 2,13-diHOME is not synthesized through DNL in BAT, but using linoleic acid, an essential fatty acid that can be obtained only from diets [127,128]. Together, the published data on adipocyte DNL suggest that DNL in WAT, but not BAT, plays an important role in the regulation of insulin sensitivity.

## 7. Role of Adipocyte DNL in Thermogenesis

In addition to the regulation of insulin sensitivity, emerging evidence indicates that DNL is also involved in thermogenesis by ATs. There are at least two types of thermogenic adipocytes, that is, brown adipocytes and cold-induced beige adipocytes, both of which are also considered to play an important role in the control of body weight and glucose homeostasis [129,130,131].

### 7.1. DNL in Thermogenesis of BAT

It is well-known that BAT plays a critical role in cold-induced thermogenesis and maintenance of euthermia [132,133]. Interestingly, although DNL in BAT is significantly induced by cold conditions, it is not essential for cold-induced thermogenesis [36]. BAT-specific loss of AKT2 disrupted ChREBP-mediated DNL in BAT, leading to impaired lipid accumulation in brown adipocytes [36]. However, those mice displayed normal heat production and the ability to maintain body temperature in response to the acute cold challenge [36]. Consistent with this study, mice with BAT-specific FASN deficiency also maintained euthermia and showed normal thermoregulation in response to cold conditions [134]. In fact, BAT lipolysis is also recently reported to be non-essential for cold-induced thermogenesis, as a result of a compensatory combustion of fuels derived from diets or lipolysis of white fat and cardiac muscle [135,136]. Thus, it seems that both lipogenesis and lipolysis in BAT are dispensable for acute cold-induced thermogenesis. However, under chronic cold adaptation, it has been shown that specific impairment of lipogenesis or lipolysis in BAT results in a compensatory response of increased WAT browning [36,136], suggesting both BAT lipogenesis and lipolysis are required for optimizing fuel storage and thermogenesis.

### 7.2. DNL in WAT Browning

The so-called WAT browning is a process in which beige adipocytes are formed in WATs in response to cold exposure or sympathetic agonist stimulation. It has several benefits, including reduction of body weight and improvement of insulin sensitivity. Recent studies indicate that DNL also plays a role in WAT browning. Contrary to the beneficial effects of adipocyte DNL described above, loss of the key lipogenic gene FASN unexpectedly stimulates the appearance of beige adipocytes in subcutaneous WAT (iWAT) in mice. When FASN is constitutively depleted in adipocytes, the mice displayed an increase in energy expenditure and cold resistance correlated with the increase of beige adipocytes in iWAT [137]. Moreover, fat-specific FASN knockout mice were protected from high-fat diet-induced obesity and exhibited an improvement of glucose tolerance and insulin sensitivity [137]. When FASN is acutely depleted in adipocytes using an inducible system, the mice displayed a significant increase in WAT browning even under a thermoneutrality condition [114]. The acute knockout of FASN in adipocytes also protected from high-fat diet-induced obesity and insulin resistance [114]. However, BAT-specific FASN deletion neither improved glucose tolerance nor induced iWAT browning [114,134], suggesting the beneficial effects of FASN deficiency in all adipocytes are only attributed to white adipocytes.

In terms of molecular mechanisms, two distinct models are proposed. In the first model, FASN is required to produce endogenous PPARγ ligands in a cell-autonomous fashion [137]. Fatty acids synthesized by FASN serve as the substrates of PexRAP, which generates alkyl ether lipids as PPARγ ligands [137]. Thus, loss of FASN decreases these ether lipids, altering the coactivator milieu to favor PPARα-dependent gene expression [137]. As a result, fatty acid oxidation and iWAT browning are increased, and diet-induced adiposity and insulin resistance are attenuated [137]. By contrast, in the second model, adipocyte FASN regulates the crosstalk between adipocytes and neurons [114,134]. In this model, FASN deficiency in adipocytes initiates neuronal signaling that triggers sympathetic stimulation to iWAT, which then activates the cAMP pathway and subsequently induces iWAT browning (Figure 2) [114,134].

Despite the disparity surrounding the molecular mechanisms, both studies show that loss of FASN indeed improves glucose metabolism and insulin sensitivity. This observation appears paradoxical to other studies showing that adipocyte DNL is beneficial to systemic glucose homeostasis and insulin sensitivity. One possible explanation is that depletion of FASN in adipocytes does not exactly mimic the decreased DNL that occurs in obesity, because other enzymes in the lipogenic pathway are also downregulated in obesity [114]

Therefore, the beneficial effects caused by the loss of FASN in adipocytes is more likely a pathophysiological compensation, instead of a physiological regulation. To better understand the role of DNL in WAT browning, adipocyte-specific ChREBP knockout mouse models may be useful in future studies.

## 8. Conclusions and Perspectives

DNL is an intrinsic metabolic process that converts excessive sugar to fat. Under normal physiological conditions, DNL in hepatocytes and adipocytes is synergistically regulated by signals from the peripheral tissues and the central nerve system. However, under pathophysiological conditions such as obesity, insulin resistance, and T2DM, the equilibration between hepatocyte and adipocyte DNL is disturbed, leading to increased DNL in the liver and decreased DNL in adipose tissues, which contributes to fatty liver and other relevant metabolic diseases.

Hepatocyte and adipocyte DNL are differentially regulated at the transcription level. SREBP-1c is a major lipogenic transcription factor in the liver, but plays a minor role in adipocyte DNL. LXRs stimulate DNL in the liver, but have almost opposite roles in adipose tissues. By contrast, ChREBPs are major lipogenic transcription factors in both hepatocytes and adipocytes. Importantly, the ChREBP levels in adipose tissues are highly correlated with insulin sensitivity in humans, and are reduced by high-fat diets or in obesity. Meanwhile, ChREBP-mediated DNL in adipocytes has beneficial effects on metabolism by generating insulin-sensitizing fatty acids such as FAHFAs. Therefore, ChREBP-mediated DNL is a potential drug target for the treatment of insulin resistance. To this end, two potential strategies may be adopted in the future. First, specific ChREBP agonists can be developed by targeting the glucose inhibitory domain or other activating steps of ChREBP-α. Second, additional beneficial products of ChREBP-mediated DNL may be identified to serve as the drug leads.

Although recent studies have provided more insights into the regulation of adipocyte DNL and its physiological relevance, there are still important questions to be addressed. Most importantly, how is DNL in adipocytes regulated differently from that in hepatocytes? The answer to this question will help us to specifically activate DNL in adipocytes. In addition, what is the physiological role of DNL in brown and beige adipocytes? Future studies on adipocyte DNL will further improve our understanding on the role of lipid metabolism in insulin resistance.

## Figures and Tables

**Table 1:** Summary of loss-of-function studies for major lipogenic transcription factors.

TF	Loss of Function	Phenotypes	References
SREBP-1	Global	Decreased hepatic lipogenesis, while increased hepatic cholesterol synthesis due to elevated SREBP-2 in liver; No effect on adiposity and lipogenic enzymes expression in WAT.	Shimano et al., 1997 [60]
	Adipose tissues	Not available	Not available
	Liver	Decreased hepatic lipogenesis, abolished sucrose-induced hypertriglyceridemia, and prevented hepatic steatosis in *ob*/*ob* mice and HFD-fed mice, despite persistent obesity, hyperinsulinemia, and hyperglycemia.	Moon, et al., 2012 [58]
ChREBP	Global	Decreased hepatic lipogenesis and glycolysis; Increased hepatic glycogen level; Reduced adiposity; Impaired insulin sensitivity and glucose tolerance.	Iizuka et al., 2004 [68]
	Adipose tissues	Decreased sucrose-induced lipogenesis in adipose tissue but not in the liver; Decreased PAHSAs level in serum; Impaired insulin sensitivity and glucose tolerance.	Vijayakumar et al., 2017 [19]
	Liver	No effects on hepatic lipogenesis, but altered expression of lipogenic genes in liver, WAT and BAT; Protected from high-carbohydrate diet induced hepatic steatosis, but with increased hepatic glucose production and impaired hepatic insulin sensitivity and systemic glucose tolerance; Reduced WAT mass and adipocyte size.	Jois et al., 2017 [89]
LXRs	Global	Decreased hepatic lipogenesis and protected from hepatic steatosis; Impaired β-cell expansion and glucose tolerance; Improved insulin sensitivity due to increased WAT lipogenesis and WAT mass.	Beaven et al., 2013 [88]
	Adipose tissues	Increased adipocyte size and adiposity by decreasing WAT lipolytic and oxidative capacities.	Dib et al., 2014 [90]
	Liver	Not available	Not available

Abbreviation: TF—transcription factor; SREBP—sterol regulatory element binding protein; ChREBP—carbohydrate response element binding protein; LXRs—liver X receptors. WAT—white adipose tissues; BAT—brown adipose tissues; HFD—high-fat diet; PAHSAs—palmitic acid ester of hydroxyl stearic acids.

**Table 2:** Summary of gain-of-function studies for major lipogenic transcription factors.

TF	Gain of Function	Phenotypes	References
SREBP-1c	Adipose tissues	Impaired adipocytes differentiation, markedly reduced adiposity; Increased fatty liver development; Impaired insulin sensitivity and glucose tolerance.	Shimomura et al., 1998 [64]
	Liver	Increased hepatic lipogenesis and fatty liver development; Increased visceral adipose tissue mass; Impaired insulin sensitivity.	Knebel et al., 2012 [56]
SREBP-1a	Adipose tissues	Increased adipose tissue lipogenesis and adipocyte hypertrophy; Enhanced fatty acid secretion and fatty liver development.	Horton et al., 2003 [63]
	Liver	Increased hepatic lipogenesis and cholesterol synthesis, and enhanced fatty liver development.	Shimano et al., 1996 [91]
ChREBP	Adipose tissues	Increased adipose tissue lipogenesis; Reduced adiposity; Protected from HFD-diet induced fatty liver; Improved insulin sensitivity and glucose tolerance.	Nuotio-Antar et al., 2015 [78]
	Liver	Increased hepatic glycolysis and lipogenesis, enhanced fatty liver development; Decreased visceral adipose tissue mass; Improved hepatic insulin signaling and systemic glucose tolerance.	Benhamed et al., 2012 [92]
LXRs	Global	Increased hepatic lipogenesis and enhanced fatty liver development; Increased WAT lipolysis and apoptosis, and decreased fat mass; Impaired insulin sensitivity but not glucose tolerance. pharmacological treatment	Dong et al., 2017 [87]

## Abbreviations

DNL	*de novo* lipogenesis
AT	adipose tissue
WAT	white adipose tissue
TG	Triglyceride
FAHFA	fatty acid ester of hydroxyl fatty acid
PAHSA	palmitic acid ester of hydroxyl stearic acid
T2DM	type 2 diabetes
CVD	cardiovascular disease
NAFLD	non-alcoholic fatty liver disease
VLDL	very low density lipoproteins
NEFA	non-esterified fatty acids
LPL	lipoprotein lipase
FATP1	fatty acid transport protein-1
ATGL	adipose triglyceride lipase
DAG	diacylglycerol
HSL	hormone-sensitive lipase
MAG	monoacylglycerol
MGL	monoacylglycerol lipase
BAT	brown adipose tissue
TCA	tricarboxylic acid
ACLY	ATP-citrate lyase
ACC1	acetyl-CoA carboxylase-1
FASN	fatty acid synthase
SREBP	sterol regulatory element-binding protein
ChREBP	carbohydrate response element-binding protein
LXR	liver X receptor
ER	endoplasmic reticulum
SCAP	SREBP cleavage-activating protein
mTORC	mammalian target of rapamycin complex
SCD1	stearoyl-CoA desaturase-1
PPAR	peroxisome proliferator-activated receptor
BCKDH	branched-chain α-ketoacid dehydrogenase
BCAA	branched-chain amino acids
AMPK	AMP-activated protein kinase
O-GlcNAc	O-Linked N-Acetylglucosamine
OGT	O-Linked N-Acetylglucosamine transferase
OGA	O-GlcNAcase
USP2A	ubiquitin-specific protease-2a
MBH	media basal hypothalamus
NPGL	neurosecretory protein GL
GPR120	G protein-coupled receptor 120
